# Range-Extension Algorithms and Strategies for TDOA Ultra-Wideband Positioning System

**DOI:** 10.3390/s23063088

**Published:** 2023-03-13

**Authors:** Shih-Ping Huang, Chien-Bang Chen, Tan-Zhi Wei, Wei-Ting Tsai, Chong-Yi Liou, Yuan-Mou Mao, Wang-Huei Sheng, Shau-Gang Mao

**Affiliations:** 1 Graduate Institute of Commutation Engineering, National Taiwan University, Taipei 106, Taiwan; 2 School of Medicine, National Taiwan University, Taipei 106, Taiwan

**Keywords:** RTLS, TDOA, ultra-wideband, extensible positioning system, out-of-range positioning

## Abstract

The Internet of Things (IoT) for smart industry requires the surveillance and management of people and objects. The ultra-wideband positioning system is an attractive solution for achieving centimeter-level accuracy in target location. While many studies have focused on improving the accuracy of the anchor coverage range, it is important to note that in practical applications, positioning areas are often limited and obstructed by furniture, shelves, pillars, or walls, which can restrict the placement of anchors. Furthermore, some positioning regions are located beyond anchor coverage, and a single group with few anchors may not be able to cover all rooms and aisles on a floor due to non-line-of-sight errors causing severe positioning errors. In this work, we propose a dynamic-reference anchor time difference of arrival (TDOA) compensation algorithm to enhance accuracy beyond anchor coverage by eliminating local minima of the TDOA loss function near anchors. We designed a multidimensional and multigroup TDOA positioning system with the aim of broadening the coverage of indoor positioning and accommodating complex indoor environments. By employing an address-filter technique and group-switching process, tags can seamlessly move between groups with a high positioning rate, low latency, and high accuracy. We deployed the system in a medical center to locate and manage researchers with infectious medical waste, demonstrating its usefulness for practical healthcare institutions. Our proposed positioning system can thus facilitate precise and wide-range indoor and outdoor wireless localization.

## 1. Introduction

### 1.1. Ultra-Wideband (UWB) Positioning

Positioning systems play a critical role in bridging the gap between people and the real world. The global positioning system (GPS) has matured in recent decades and has proven to be reliable technology for outdoor navigation, disaster rescue, and aircraft stabilization [1]. However, GPS is not suitable for indoor environments. Indoor positioning is vital for surveillance and management applications, including guiding customers in a shopping mall, navigating autonomous guided vehicles (AGVs) in factories or warehouses [2,3], developing collision avoidance systems for AGVs [4], and managing hazardous materials in a laboratory. A summary of recent indoor positioning scenarios and technologies is presented [5].

One commonly used technology for achieving high-accuracy positioning systems is ultra-wideband (UWB). Well known mobile phone providers, such as Apple [6], Google [7], and Samsung, develop their products with UWB chips [8]. For example, Apple’s AirTag, released in 2021, uses UWB technology to assist iPhone users in locating lost items [9]. UWB signals are impulses in the time domain with a bandwidth greater than 500 MHz. The precise impulse timestamps can help reduce the multipath effect. UWB chips provide channel impulse responses (CIRs), which can be used for non-line-of-sight (NLOS) identification or mitigation [10,11,12]. UWB can be combined with inertial measurement units (IMU) and GPS for indoor–outdoor roaming [13,14].

### 1.2. Time of Arrival (TOA)

Time of arrival (TOA) is the predominant positioning method employed in most UWB positioning systems. The distance between anchors and tags can be determined using two-way-ranging (TWR) [15]. Precise distance measurement enables accurate estimation of both 2D and 3D positions [16,17,18]. While the accuracy of TOA is generally superior among time-based methods, the latency of target positioning tends to increase with an increasing number of tags [19]. Consequently, the localization rate decreases rapidly, rendering TOA unsuitable for applications that involve a large number of targets.

### 1.3. Time Difference of Arrival (TDOA)

The time difference of arrival (TDOA) method estimates the differences in distance between a tag and any two anchors and then identifies the intersection of the hyperbolas to locate the tag. In an uplink TDOA system, anchors are synchronized using Ethernet or wireless signals [20]. Once the tag sends a data frame, the time differences can be computed. By using the ALOHA theorem [21], only approximately 18.6% of the timeframe can be utilized for collision-free communication because tags are not scheduled and transmitted randomly. Thus the tags can conserve power and reduce costs because random transmission does not require a powerful microcontroller.

In the downlink TDOA system, the slave anchors are triggered by a master anchor to transmit ranging messages in scheduled slots. The tags can determine the differences in the distance by comparing the receiving timestamps of the ranging messages [22]. The number of tags in the downlink system is unlimited, as the tags do not need to transmit messages. The positioning result is computed on the tag and must be uploaded to the server by out-of-band protocols such as Bluetooth low energy (BLE) or WiFi. Fortunately, the targets already equipped with such commercial wireless protocols can be quickly integrated with a downlink TDOA system. For example, AGVs are often monitored by WiFi, and mobile phones can access the Internet via 4G or 5G communication.

### 1.4. Extended Positioning Requirements

The positioning accuracy within the convex hull of the anchors surpasses that outside [23]. Ideally, anchors should be deployed to cover all desired positioning areas. However, realization and power concerns may prohibit anchor placement in the desired locations, resulting in parts of the positioning area being outside the convex hull. Additionally, in specific applications, such as drone positioning and landing systems [24,25,26], the target may be far from the anchors. The validation of out-of-range positioning is crucial for implementing and extending the usage of a positioning system.

### 1.5. Contributions

This study proposes a dynamic-reference anchor (DRA) algorithm to improve TDOA out-of-range positioning accuracy. The local minima in the loss function can be eliminated by selecting a proper reference anchor. In addition, a multidimensional and multigroup TDOA system is designed to expand the positioning coverage. The major contributions of this study are summarized as follows:

- The DRA algorithm eliminates the local minima in the loss function, improving the out-of-range accuracy of TDOA.
- Positioning coverage can be expanded by using multiple individual groups, and the group searching algorithm minimizes the roaming latency of tag.
- The multidimensional groups are versatile in adapting to various complex indoor environments.

This paper is organized as follows. Section 2 provides a comparison of this proposed work with other related works. Section 3 analyzes the problem of the TDOA loss function outside the coverage of anchors. A simulation and experiment are conducted to prove the improvement of the DRA algorithm. Section 4 presents the multigroup and multidimensional TDOA positioning system, including group types, group bounding, group region, and the process of group switching. In Section 5, the proposed system is demonstrated with an application of medical waste management in a medical center. The bias problem of the 1D group is discussed in Section 6.1, and a solution is given in Section 6.2. Finally, Section 7 summarizes the conclusions and outlines future work.

## 2. Related Works

A system that uses additional anchors to expand the positioning range was proposed [27]. When the positioning target moves to unexpected areas, a new anchor can be deployed and located by the original anchors. However, the estimation errors of new anchors may accumulate when more additional anchors are used.

Ref. [28] used a positioning target with two tags, each connected to a different UWB network. The fusion of ranging results can improve accuracy if the quality of ranging is poor in one of the UWB networks. Although the concept of fusing multiple tags is interesting, it is not practical to equip a small device with a large number of tags in multiple networks.

The out-of-range positioning scenario in smart-following applications was discussed [29,30]. When the tag carried by a human master moves far away, the anchors on an AGV concentrate like a hotspot. The proposed anchor selection method uses the most asymmetric anchors to locate the tag to improve accuracy. However, in a rectangular room, the acceptable number of anchors is usually at most four, and only a few subsets can be selected.

Despite numerous studies on TDOA single-group wireless synchronization using stationary anchors [31,32,33], few practical solutions have been proposed to extend positioning coverage in severe NLOS scenarios. The plug-and-play localization system (PnPLoc) in which all anchors initiate TWR with nearby anchors was proposed [34], and additional anchors are not limited by the distance from a specified master anchor. However, the positioning rate decreases when there are many anchors in a large building. A multi-hop wireless synchronization algorithm was proposed in [35], where the synchronization signal from the master anchor propagates between anchors according to the synchronization quality treemap, reducing the synchronization error caused by NLOS in a building. However, line-of-sight (LOS) anchor pairs may not always be present between adjacent rooms, and a viable path may not be included in the treemap. The study in [36] proposed a grid-based positioning system in which TDOA anchors are placed as multiple grids and synchronized using wireless signals. Every anchor establishes a connection to the Internet to schedule time-division multiple access slots and transmit positioning data, while tags connect to the cell with the strongest received signal strength. Nevertheless, the proposed grid-like anchor deployment is unfeasible for complex indoor environments.

## 3. TDOA Positioning Algorithm

### 3.1. Existing Solutions

**Figure 1:**
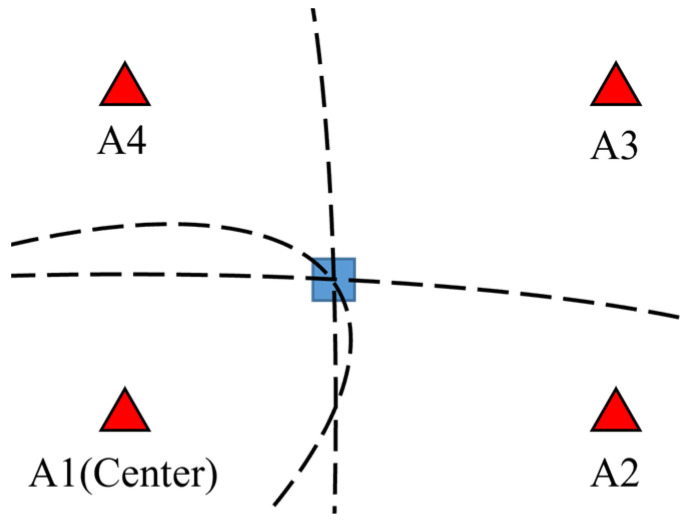
Time difference of arrival (TDOA) hyperbolas. The blue square denotes the tag.

Consider a TDOA network with four anchors and a tag. The measured signal time difference between the ith anchor and the jth anchor, denoted as $t_{ij}$, can be translated to the distance difference $d_{ij}$ by multiplying it by the light speed. Because $d_{ij}$ can be obtained from $d_{ik}-d_{jk}$, any anchor can be chosen as the reference anchor. For example, if k = 1, the linear independent terms are $d_{21}$, $d_{31}$, and $d_{41}$. The hyperbolas shaped by these terms would intersect at the tag position, as depicted in Figure 1.

In practical scenarios, hyperbolas often do not intersect at a single point due to measurement errors. Several TDOA algorithms have been proposed to find the optimal position, including the least square (LS) closed-form solution [37], Chan method [38], Taylor method [39], and gradient descent (GD) algorithm. However, LS and Chan methods may not provide sufficient accuracy due to approximation errors, while the Taylor method may converge to an incorrect position if the initial estimate is far from the true position. Although the GD algorithm provides better accuracy, it requires heavy computations. A hybrid method that combines Taylor and GD methods was proposed in [38] to improve accuracy, but it is still unsuitable for cost-effective IoT devices. A second-order term was added to the Taylor series [40] to improve the Taylor method, which achieved better convergence performance than the traditional Taylor method. In this study, we adopted the second-order Taylor (SOT) method as the base TDOA optimization algorithm. A brief derivation of SOT is presented below.

Suppose anchor k is selected as the reference anchor. The estimated position is $p_{v}=[x_{v}\;y_{v}]$ with arbitrary initial values, and the number of anchors is N. The loss function at position p=[x y] to the ith anchor and the reference anchor can be defined as

(1) $$f_{i}\left(p\right)=\sqrt{{\left(x_{i}-x\right)}^{2}+{\left(y_{i}-y\right)}^{2}}-\sqrt{{\left(x_{k}-x\right)}^{2}+{\left(y_{k}-y\right)}^{2}}=r_{i,k}+\epsilon_{i,k},$$

where $r_{i,k}$ is the measured distance difference and $\epsilon_{i,k}$ is the measurement error. By expanding (1) using Taylor series at $p_{v}$ and ignoring the high-order terms, (1) can be rewritten as

(2) $$f_{i}\left(p_{v}\right)+a_{i}\delta ≅r_{i,k},\;i=1,2,\ldots ,N,$$

where

$$a_{i}=\left[\begin{array}{cc} \frac{\partial f_{i}\left(p\right)}{\partial x} & \frac{\partial f_{i}\left(p\right)}{\partial y} \end{array}\right],\delta =\left[\begin{array}{c} x_{v}-x\\ y_{v}-y \end{array}\right].$$

Then, the first-order Taylor step $\delta_{1}$ can be solved as:

(3) $$\delta_{1}={\left({A_{1}}^{T}A_{1}\right)}^{-1}{A_{1}}^{T}D,$$

where

$$A_{1}={\left[\begin{array}{cccc} a_{1} & a_{2} & \cdots & a_{N} \end{array}\right]}^{T},D=\left[\begin{array}{c} r_{1,1}-f_{1}\left(p_{v}\right)\\ r_{2,1}-f_{2}\left(p_{v}\right)\\ \vdots \\ r_{N,1}-f_{N}\left(p_{v}\right) \end{array}\right]$$

Now consider the second-order term in the Taylor series; (2) becomes:

(4) $$f_{i}\left(p_{v}\right)+a_{i}\delta +\frac{1}{2}\delta^{T}H_{i}\delta ≅r_{i,1},\;i=2,3,\ldots ,N,$$

where

$$H_{i}=\left[\begin{array}{cc} \frac{\partial^{2}f_{i}\left(p\right)}{\partial x^{2}} & \frac{\partial^{2}f_{i}\left(p\right)}{\partial x\partial y}\\ \frac{\partial^{2}f_{i}\left(p\right)}{\partial x\partial y} & \frac{\partial^{2}f_{i}\left(p\right)}{\partial y^{2}} \end{array}\right].$$

Because $\delta_{1}$ is determined, which can be regarded as a constant, the second-order term $\delta^{T}$ can be approximated by ${\delta_{1}}^{T}$ to translate (4) into a linear equation

(5) $$f_{i}\left(p_{v}\right)+(a_{i}+\frac{1}{2}{\delta_{1}}^{T}H_{i})\delta ≅r_{i,1},\;i=2,3,\ldots ,N.$$

The second-order step $\delta_{2}$ can be solved by the least squares estimator

(6) $$\delta_{2}={\left({A_{2}}^{T}A_{2}\right)}^{-1}{A_{2}}^{T}D,$$

where

$$A_{2}=A_{1}+\frac{1}{2}{\left[\begin{array}{cccc} {H_{1}}^{T}\delta_{1} & {H_{2}}^{T}\delta_{1} & \cdots & {H_{N}}^{T}\delta_{1} \end{array}\right]}^{T}.$$

Finally, $p_{v}$ can be updated by adding $\delta_{2}$ at each iteration until $\delta_{2}$ is smaller than the early stopping criterion, or the maximal iteration is reached. The derivation of SOT in detail can be found in [40].

### 3.2. Performance Analysis

**Figure 2:**
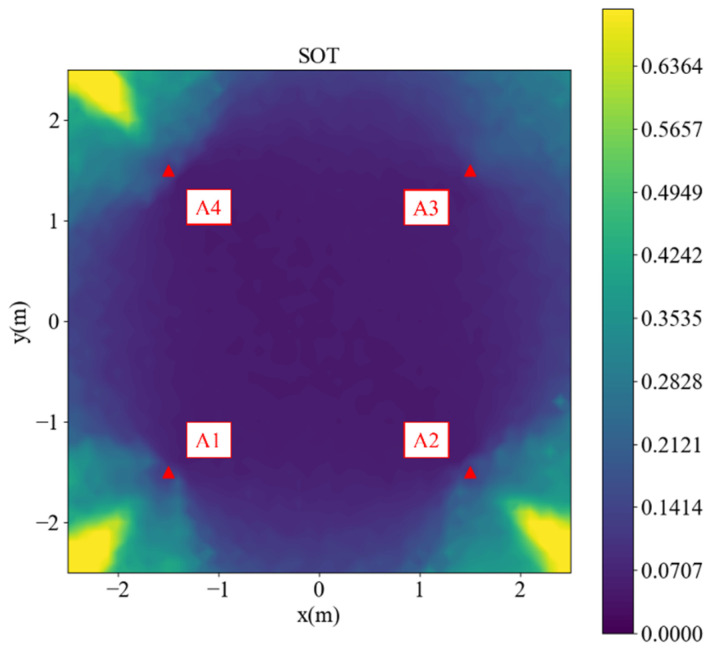
Simulated root-mean-square error (RMSE) of the second-order Taylor (SOT) method.

A simulation of the SOT was performed for both the inside and outside areas. The simulation range was 3 m × 3 m, and the grid size was 0.1 m, where each point took 100 tests. In Figure 2, the red dots located at (−1.5, −1.5), (1.5, −1.5), (1.5, 1.5), and (−1.5, 1.5) are anchors 1 to 4, respectively. Anchor 1 was selected as the reference. Because there was no prior knowledge of the correct position, the estimated initial position was at the center of the anchors (0.0, 0.0) in each test. The simulated distance differences were noised by σ~N(0, 0.06). The maximum epoch of the SOT was 30, and the early stop criterion was that the 2-norm of $\delta_{2}$ was smaller than 0.001 m. The simulated root-mean-square error (RMSE) is shown in Figure 2. Values are represented by colors in the color bar. The areas with an RMSE greater than 0.7 m are colored as 0.7 m for better visualization. 

**Figure 3:**
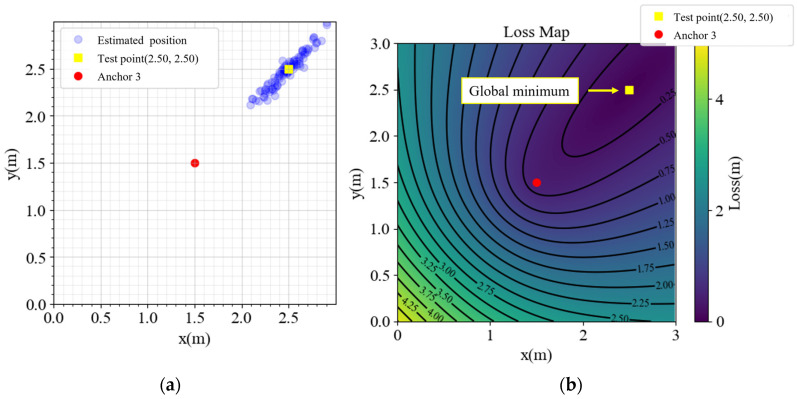
(**a**) Estimated positions and (**b**) loss map of point (2.5, 2.5).

**Figure 4:**
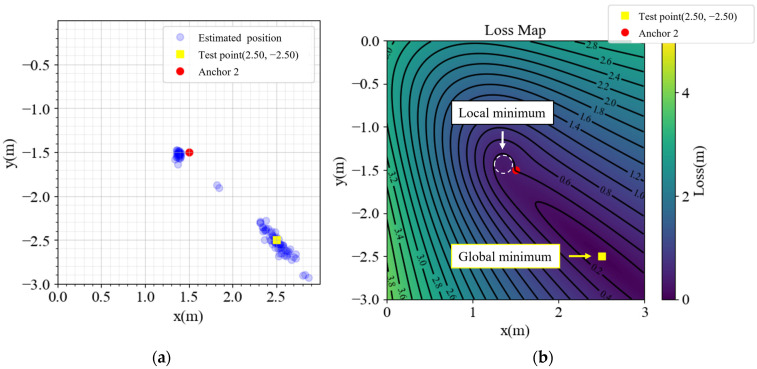
(**a**) Estimated positions and (**b**) loss map of point (2.5, −2.5).

**Figure 5:**
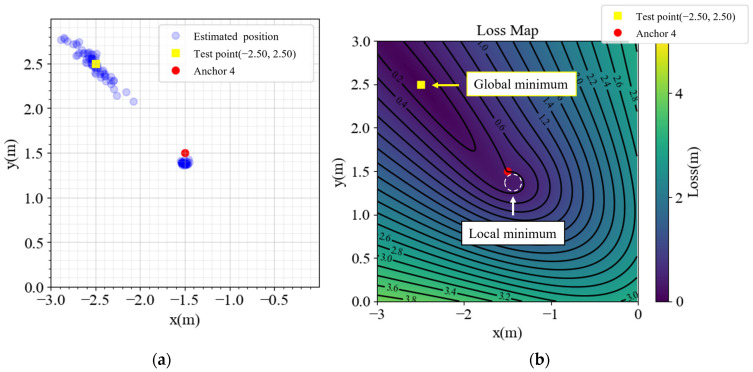
(**a**) Estimated positions and (**b**) loss map of point (−2.5, 2.5).

**Figure 6:**
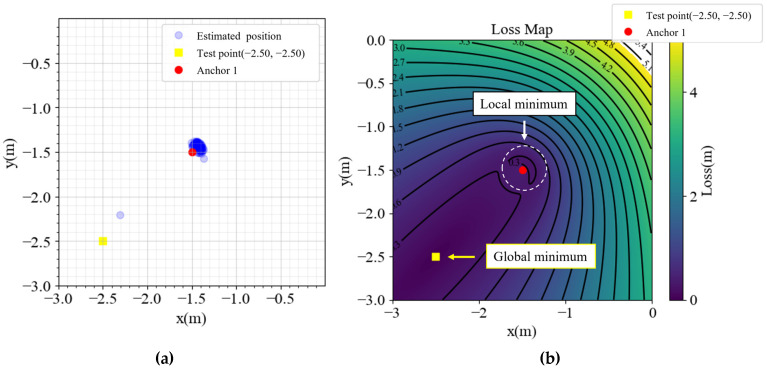
(**a**) Estimated positions and (**b**) loss map of point (−2.5, −2.5).

In Figure 2, the performance is satisfactory inside the anchors, but outside the area, especially in the yellow cones outside anchors 1, 2, and 4, the performance is not optimal. Detailed analyses of the points (2.5, 2.5), (2.5, −2.5), (−2.5, 2.5), and (−2.5, −2.5) are presented in Figure 3, Figure 4, Figure 5 and Figure 6, respectively, to investigate why the yellow cone disappears outside anchor 3. In Figure 3a, the estimated positions are scattered around the actual position. However, as depicted in Figure 4a, the positions are split into two clusters. The cluster close to anchor 2 might be trapped in the local minimum of the loss map, as demonstrated in Figure 4b. The loss map was computed using noiseless data. However, due to noise in actual data, there could be changes in the loss map, causing estimations to converge towards local or global minima. A similar phenomenon is observed in Figure 5 and Figure 6.

In contrast, Figure 3b exhibits no local minima. The TDOA hyperbolas are responsible for the absence of local minima on the opposite side of the reference anchor. Although different choices of the reference anchor should yield the same global minima, the local minima positions depend on the reference anchor.

### 3.3. Dynamic Reference Anchor Algorithm

Because the opposite side of the reference anchor performs better than the others, the reference anchor can be changed according to the estimated position. This study proposes a dynamic reference anchor second-order Taylor (DRA-SOT) algorithm that updates the reference anchor in each iteration to eliminate the local minima that appear at the outside corner.

Suppose anchor 1 is the reference anchor during the measurement process. The original anchor position matrix A can be defined as

(7) $$A=\left[\begin{array}{c} \begin{array}{cccc} p_{1} & p_{2} & \ldots & p_{N} \end{array} \end{array}\right],$$

where $p_{n}$ is the position of the nth anchor, and the first term is the reference anchor. The measured distance difference vector R can be defined as

(8) $$R=\left[\begin{array}{c} \begin{array}{cccc} r_{1,1} & r_{2,1} & \ldots & r_{N,1} \end{array} \end{array}\right],$$

where $r_{i,j}$ is defined as in Equation (1). Because there is no prior knowledge of the correct position, the reference anchor is arbitrary in the first iteration and the initial position $p_{v}$ can be set to the center of the anchors. In the second iteration, as $p_{v}$ moves toward the target, the index of the farthest anchor to $p_{v}$ can be found by

(9) $$\alpha =\underset{i}{\mathrm{argmax}}\sqrt{{\left(x_{i}-x_{v}\right)}^{2}+{\left(y_{i}-y_{v}\right)}^{2}}.$$

Then, the translated data vector $R^{\prime }$ can be generated by subtracting R by $r_{\alpha ,1}$ to make the arguments referenced to anchor α. For example, if N=4 and α=3, then

(10) $$R^{\prime }=\left[r_{1,1}\begin{array}{c} \begin{array}{cccc} {-r}_{3,1} & r_{2,1}{-r}_{3,1} & 0 & r_{4,1}{-r}_{3,1} \end{array} \end{array}\right].$$

The first term of $R^{\prime }$ is the reference distance difference in the SOT method. Thus, $R^{\prime }$ is updated by exchanging $R^{\prime }[0]$ and $R^{\prime }[\alpha ]$:

(11) $$R^{\prime }=\left[\begin{array}{c} \begin{array}{cccc} 0 & r_{2,1}{-r}_{3,1} & r_{1,1}{-r}_{3,1} & r_{4,1}{-r}_{3,1} \end{array} \end{array}\right].$$

In addition, the anchor position matrix is translated by exchanging A[0] and A[α]:

(12) $$A^{\prime }=\left[\begin{array}{c} \begin{array}{cccc} p_{3} & p_{2} & p_{0} & p_{4} \end{array} \end{array}\right].$$

Finally, $A^{\prime }$ and $R^{\prime }$ are passed to the SOT to update $p_{v}$ at each iteration. Note that the term $r_{i,j}$ for which i is equal to j is always 0. The procedure of DRA-SOT is shown in Algorithm 1.
**Algorithm 1** Function DRA_SOT()**Input**Anchor positions: ($x_{1}$, $y_{1}$), ($x_{2}$*,*
$y_{2}$), …, ($x_{N}$*,*
$y_{N}$)Distance differences: $r_{1,1}$, $r_{2,1}$, …, $r_{N,1}$Early stop criterion: βMaximal iteration: *max_epoch***Output**Estimated position: ($x_{est}$, $y_{est}$)1Set (*x*, *y*) to the center of anchors2Set *n* to 03**while***n* < *max_epoch* **do**4 use (9) to find the reference anchor’s index α5 Set R to [$r_{1,1}$, $r_{2,1}$, …, $r_{N,1}$] - $r_{\alpha ,1}$6 Set A to [[$x_{1}$, $y_{1}$], [$x_{2}$, $y_{2}$], …, [$x_{N}$, $y_{N}$]]7 Exchange R[0] for R[α]8 Exchange A[0] for A[α]9 Use (3) to determine $\delta_{1}$10 Use (6) to determine $\delta_{2}$11 Set (x,y) to (x + $\delta_{2x}$, y + $\delta_{2y}$)12 Set *n* to *n* + 113 **if** norm($\delta_{2}$) < β **then**14  **break**15 **end if**16**end while**17**return** (*x*, *y*)

### 3.4. Simulation

**Figure 7:**
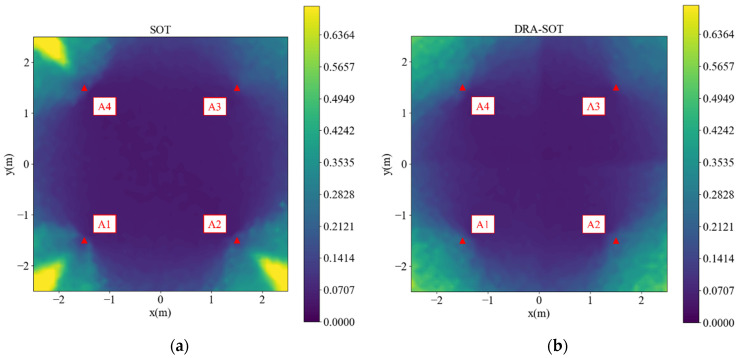
Simulated RMSE for square deployment using (**a**) SOT, (**b**) dynamic reference anchor SOT (DRA-SOT).

Figure 7a is a copy of Figure 2 and is placed here for comparison. The simulation results of DRA-SOT are plotted in Figure 7b. The ranges and conditions were the same as those described in Section 3.2. Compared with Figure 7a, the yellow cones outside the anchors disappear, while the other areas do not change much. The accuracy was significantly improved. 

**Figure 8:**
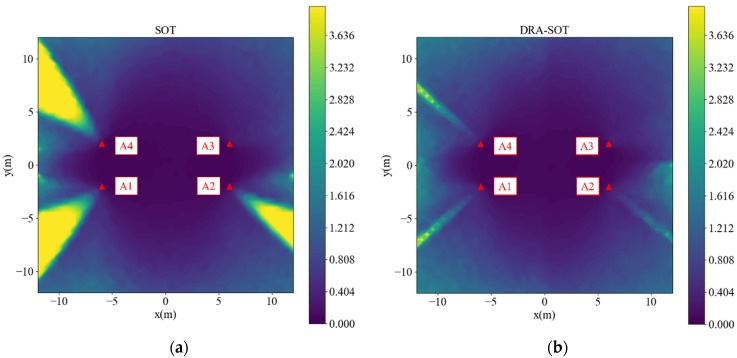
Simulated RMSE for rectangle deployment using (**a**) SOT, (**b**) DRA-SOT.

Another simulation of a rectangular case is performed, as shown in Figure 8. As in the square case, yellow cones occur at the outside corners in Figure 8a using the SOT method, except for anchor 3. The DRA-SOT method eliminates most areas of the yellow cones, as shown in Figure 8b. 

### 3.5. Experiment

**Figure 9:**
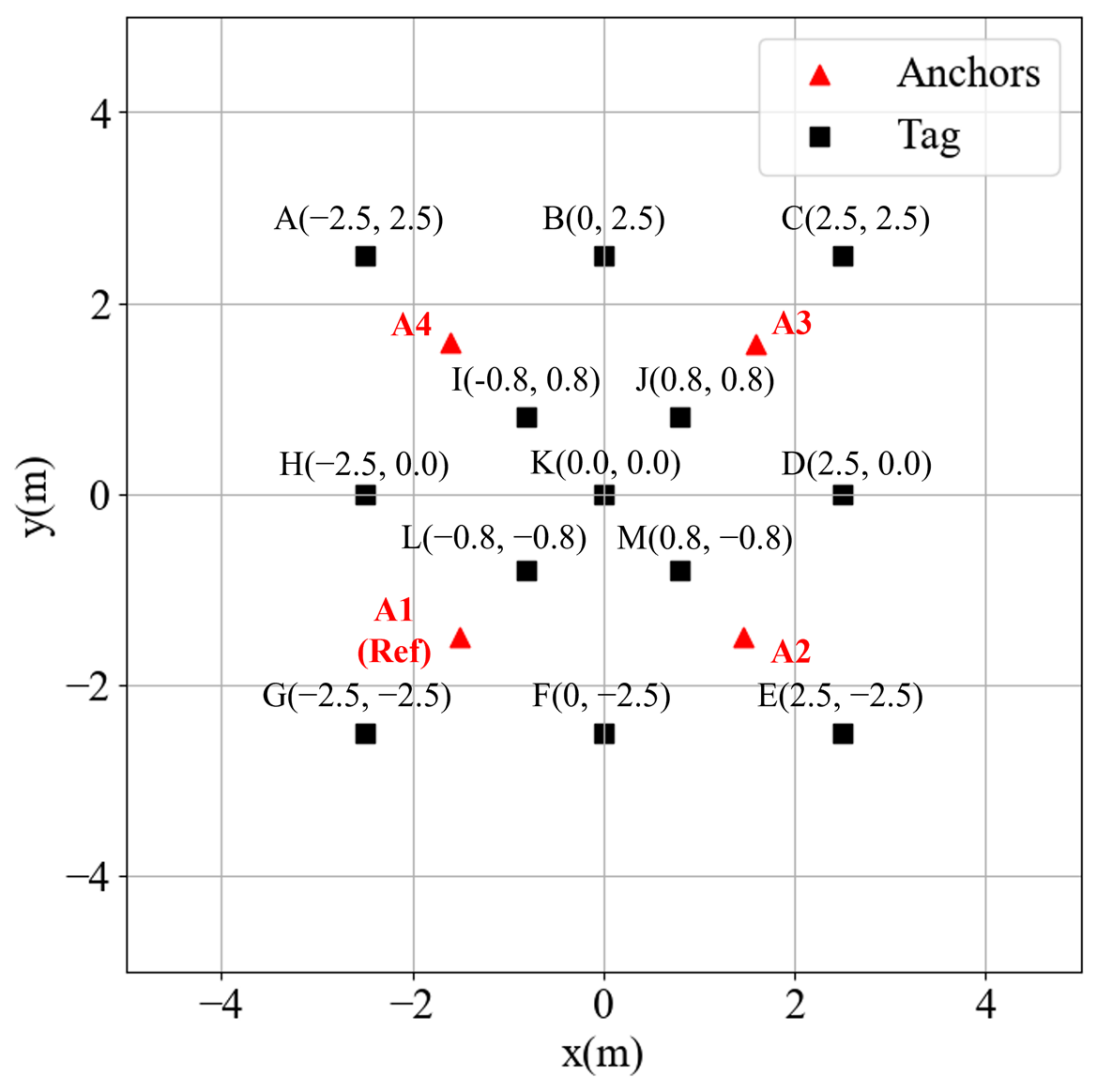
Testing points.

**Figure 10:**
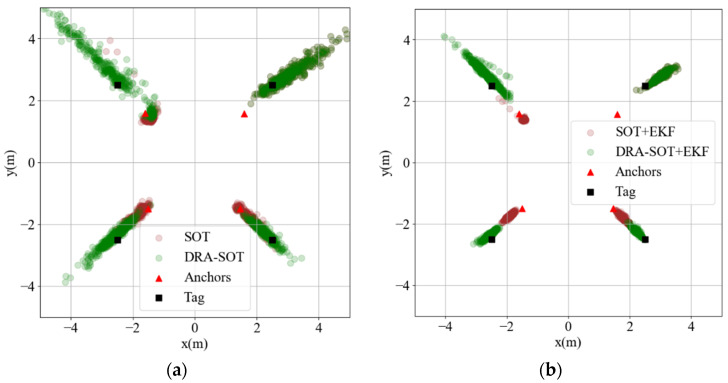
Experiment results (**a**) without extended Kalman filter (EKF), (**b**) with EKF.

In the experiment, the anchors were placed at (±1.5 m, ±1.5 m), and the testing points are plotted in Figure 9 The tag collected 400 TDOA data points for each testing point and computed the position using the SOT and DRA-SOT methods. The reference anchor of SOT is anchor 1. The positioning results for the outside corner points A, C, E, and G are analyzed in Figure 10a. The red triangles denote the anchors. The brown and green dots represent the estimated positions using SOT and DRA-SOT, respectively. Most DRA-SOT dots are distributed around the correct position, whereas the SOT dots are often stuck at the local minima near the anchors, as in the simulation. The upper-right corner shows only green dots because the corner is already on the opposite side of the default reference anchor, and thus the estimations using SOT and DRA-SOT fully overlap. 

Although the DRA-SOT method overcomes the local minima problem, the dots are scattered and spread. An effective strategy to stabilize these positions is to adopt an extended Kalman filter (EKF). The EKF is a predictor based on the Markov chain, which assumes that the positioning target obeys physical principles. It has been widely used in sensor fusion and data filtering [41,42,43]. The positions of the applied EKF are shown in Figure 10b. The SOT dots are still stuck at the local minima because filtering cannot eliminate bias. In contrast, the DRA-SOT dots become more centralized in the correct location.

The positioning RMSEs of all testing points are listed in Table 1. The outside corner points A, C, E, and G are significantly improved. The accuracy of internal points I, J, K, L, and M and the outside-edge points B, C, D, F, and H are almost the same for both methods. It was proven that the proposed DRA-SOT method with EKF could effectively improve the accuracy outside the range of anchors for a TDOA positioning system.

## 4. Multi-Group and Multi-Dimensional TDOA Network

### 4.1. Motivation

**Figure 11:**
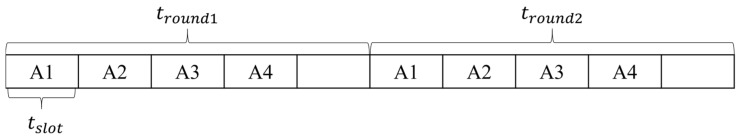
Slots of ranging rounds.

In this study, a TDOA positioning network consisting of a master anchor and a few slave anchors was called a group. The communication process can be found in [22]. The master anchor, usually anchor 1, schedules message slots for all slave anchors, as illustrated in Figure 11. When the slave anchors receive a control message from the master anchor, they transmit ranging messages at each scheduled time. In this network, the last anchor slot is followed by an empty slot to allow the tags to compute the positions. Assuming that the slot duration is $t_{slot}$ and the number of anchors is N, the period of a ranging round is

(13) $$t_{round}=(N+1)t_{slot}$$

where an additional 1 represents an empty slot.

The clock difference between a tag and anchor may result in timestamp errors. This error can be compensated for by the time ratio of the transmitting period of an anchor and the receiving period at the tag in two ranging rounds [44]. Thus, a tag has to receive two ranging rounds to compute the corrected time difference.

Although the UWB positioning system performs well for a single group in an ideal environment, it is still unsuitable for indoor environments with rooms, walls, and pillars because of severe NLOS errors [33,45]. The impact of large metal objects is even more significant [46]. In addition, the anchors can be saved in an aisle-like environment when only a 1D position is required. A multigroup and multidimensional seamless TDOA network is proposed in this section to accommodate various indoor environments and positioning requirements.

### 4.2. Group Type

- 2D Group

A group requires at least two hyperbolas to find the intersection point as the 2D position of the tag. Because the TDOA between two anchors shapes only one hyperbola, at least three anchors are required in a 2D group. The DRA-SOT algorithm introduced in Section 3.3 can be applied.

- 1D Group

Most indoor environments include rooms and aisles. The 2D positioning system can be used in room-like spaces, but it may not be suitable for aisles. The geometric dilution of precision (GDOP) was developed to estimate the accuracy of satellite positioning and can be applied to a UWB positioning system [47]. According to [48], the GDOP of 2D anchors in a narrow aisle is excessively high, resulting in inaccurate estimations perpendicular to the aisle. 

**Figure 12:**
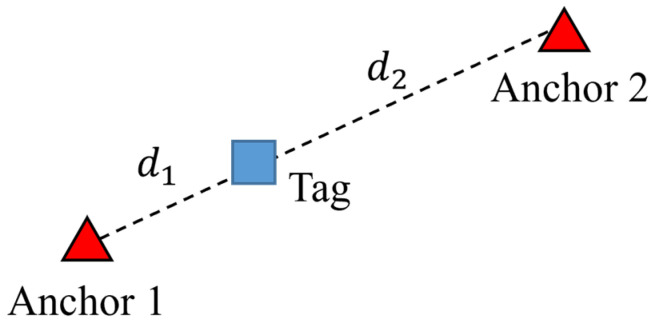
The 1D group structure.

A 1D group aims to find the tag’s position between the two anchors, as depicted in Figure 12. The cost of manufacturing and deployment of anchors can be reduced when only the 1D positions of tags are required. Let us assume that the positions of anchors 1 and 2 are $p_{1}$ and $p_{2}$, respectively. The tag is positioned at $p_{v}$. The distances between the two anchors and the tag are $d_{1}$ and $d_{2}$. Then, $p_{v}$ can be solved as follows:

(14) $$p_{v}=p_{1}+\frac{d_{1}}{norm(p_{1},p_{2})}(p_{2}-p_{1}).$$

### 4.3. Group Bounding

Messages from different groups may be unaligned or conflicting. The package loss increases as the number of groups increases. Although the fully conflicting messages of different groups may not be received, the tag can minimize the processing time after receiving a message and restart receiving to capture more nearby messages.

The ranging message contains the group ID of each anchor. Because the tag needs only one group at a time to find its position, the idea is to select an appropriate group to bind and discard the messages from the other groups. When the tag receives a ranging message, it first checks whether the group ID is that expected. If so, it extracts the timestamp and calculates TDOA. Otherwise, it directly filters the message and restarts receiving it to save processing time.

**Figure 13:**
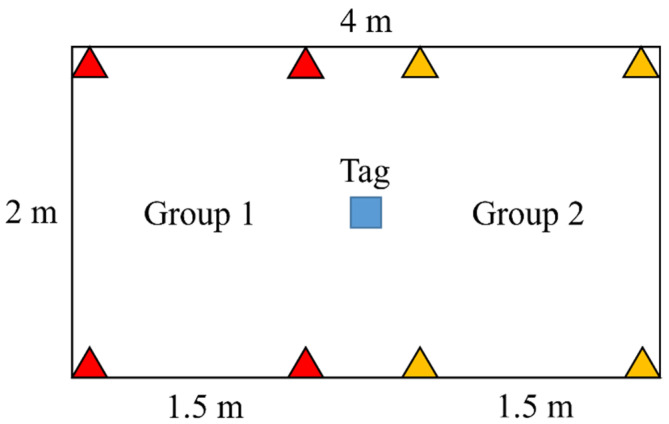
Group filtering deployment.

An experiment was conducted to verify the benefits of group filtering. Two groups and one tag are placed in a laboratory, as shown in Figure 13. The tag was located at the center of each group. The slot duration was 15 ms in each group. The ranging period was 75 ms, calculated using (13). The ideal number of successful positions was 799 in 1 min, where the first round was excluded. The tag recorded the number of successful positions for 1 min when binding to group 1, to group 2, and without binding. Successful positioning can be considered only when the tag receives all messages in two consecutive rounds. The testing results are shown in Table 2 and Table 3. When no group was bound, the successful positioning percentages were 35.2% and 39.9% in each group, respectively, with an average of 37.55%. The percentages in the binding cases were 85.7% and 76.0%, respectively, with an average of 80.85%. This shows that binding to a group can significantly increase the probability of successful positioning in a multigroup network.

### 4.4. Group Region

#### 4.4.1. Region Definition

**Figure 14:**
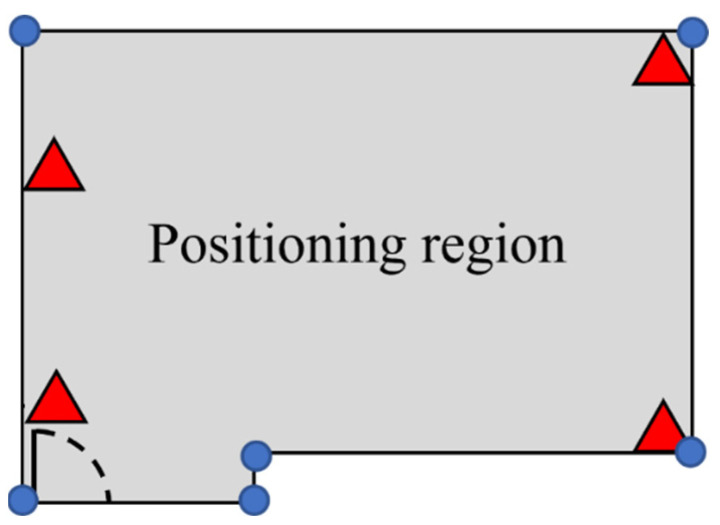
Region of a 2D group.

The tag must know the effective positioning region of a group to bind or unbind it. The ideal positioning region is the convex hull of the anchors that covers all positioning areas. However, anchors may not be allowed to be placed at ideal locations. The positioning environment is not always a perfect rectangle that the four anchors can easily cover. The positioning region of a group should be defined according to the actual scenario. An example is shown in Figure 14. The red triangles represent anchors, and the gray area represents the desired positioning area. Blue points indicate the positioning regions. 

#### 4.4.2. Ray-Casting Algorithm 

**Figure 15:**
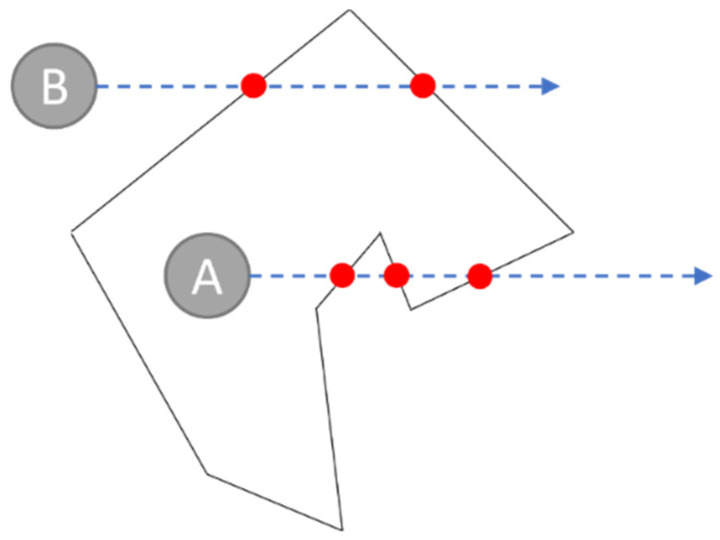
The ray-casting algorithm.

The positioning region is always a simple polygon in which any edge would never cut the other edge [49]. The ray-casting algorithm [50] can be used to check whether the Tag’s position is inside or outside a region. If a point lies inside a simple polygon, the ray cast from the point in any direction will intersect the polygon an odd number of times. An intersection implies an inside–outside state exchange. Because the ray will eventually be out of the polygon, the intersection number would always be odd if the point is inside. An example is shown in Figure 15. The ray cast from point A intersects with the edges an odd number of times, while that from point B intersects an even number of times.

**Figure 16:**
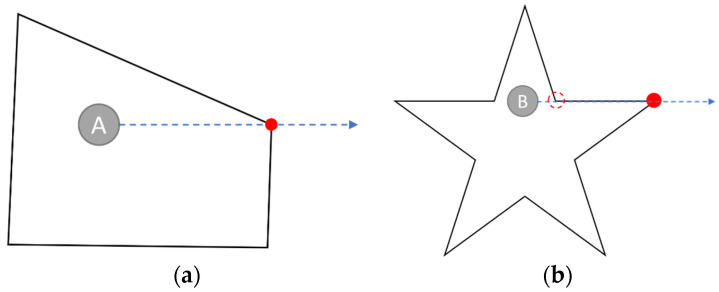
The (**a**)common terminal case (**b**) line segment case of polygon intersection.

Suppose the ray is cast toward the right. There is no intersection if a line segment is totally above or below the test point or lies on the left plane of the point. The critical cases are shown in Figure 16. In Figure 16a, the intersection should be considered only once when the ray starts from the point A passes through the common terminal of two line segments. Therefore, it should not be considered an intersection if the lower point of a segment touches the ray. In Figure 16b, the red-dashed circle represents a lower point that is ignored, and the horizontal line segment does not intersect with the ray cast from point B.

The ray-casting algorithm is implemented in Algorithm 2. Line 8 filters the cases when the segment is above *p*, the segment is horizontal, and the lower point of the segment touches the ray. Line 10 filters the case that the segment is on the left of the point. Lines 13–15 compute the slope of the segment and check if the intersection is on the left or right.
**Algorithm 2** Function is PointWithinPolygon()**Input**Test point *p*Polygon points $s_{1}$, $s_{2}$, …, $s_{N}$, $s_{1}$**Output**Verified result *result*1**Set** *count* **to** 02**Set** *i* **to** 03**while** *i* < N **do**4 **Set** ($p_{start},\;p_{end}$) **to** (*s*[*i*], *s*[*i* + 1])5 **Set**
*i*
**to**
*i* + 1 6 **if**
$p_{start}$[1] > = *p*[1] and $p_{end}$[1] > = *p*[1] **then**7 **continue**8 **else if**
$p_{start}$[0] < *p*[0] and $p_{end}$[0] < *p*[0] **then**9  **continue**10 **end if**11 **Set**
*m*
**to** ($p_{end}$[0] − $p_{start}$ [0])/($p_{end}$ [1] − $p_{start}$[1])12 **Set**
$x_{cross}$
**to**
$p_{end}$[0] − ($p_{end}$[1] − *p*[1]) × *m*13 **if**
$x_{cross}$ < *p*[0] **then**14 **continue**15 **end if**16 **Set**
*count* **to**
*count* + 117**end while**18**if** *count* is odd **then**19 **return** True20**end if**21**return** False

### 4.5. Group Switching

#### 4.5.1. Process

**Figure 17:**
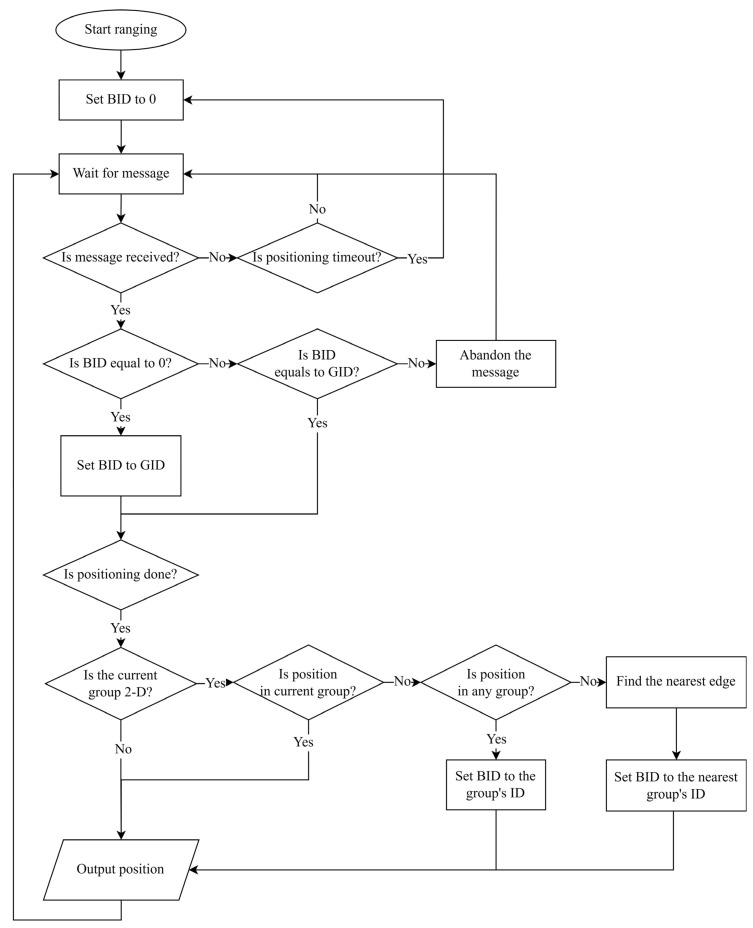
Process of switching group.

A diagram of the group switching is shown in Figure 17. The parameter GID represents the group ID of the received message. The currently bound group ID is denoted as BID. Note that the group IDs start from 1, where 0 is reserved for the no-bound case.

The BID value remains unchanged if the position is within the current group’s region. Otherwise, the tag searches for the group in the database whose region includes the position and assigns the BID to its ID, prioritizing 2D groups over 1D groups. If the tag does not fall within any group’s region, it assigns the BID to the nearest group’s ID based on the nearest boundary.

If the tag loses signal or cannot receive messages for a period, it should set the BID to 0 and release its association with the current group. To avoid message conflict when there is no existing group association, the tag may establish a binding with the first group it receives when the BID value is 0 until it obtains a position or times out.

The TDOA-based estimated position in 1D groups is always between the anchors and cannot determine whether the tag is inside or outside the region. However, 1D groups can be used in indoor aisle-style environments with concrete walls on both sides. If the tag leaves the aisle, the signal decays rapidly, and the tag can stop binding to the aisle group and be considered a positioning timeout case.

#### 4.5.2. Latency

**Figure 18:**
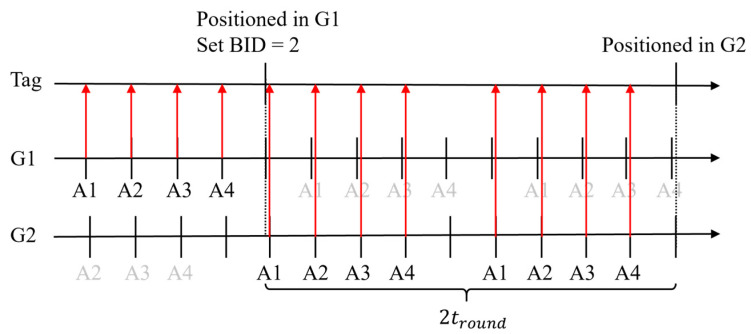
The best case for the switching group.

**Figure 19:**
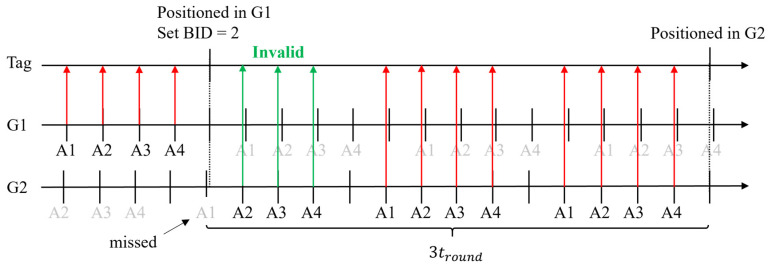
The worst case for the switching group.

Let us assume a tag is positioned in group 1, but the position lies in group 2. After calculating the position, according to the rule of group switching, the tag changes the BID to 2. If the message from anchor 1 in group 2 is received immediately after changing the BID, then the latency would be two ranging periods, as shown in Figure 18. However, if anchor 1 of group 2 coincidentally transmits before the tag changes the BID, the message will be missed, as shown in Figure 19. The other messages in this ranging round will be invalid, and the latency will be three ranging periods.

Consider the probability of successful positioning discussed in Section 4.3. The expected latency of the best case can be corrected as

(15) $$t_{sw\_best}=\frac{{2t}_{round}}{R},$$

where R, is the successful positioning percentage which is 80.85%. In the worst case, the first ranging period does not need to be corrected because missing invalid messages are unimportant. The expected latency of the worst case can be corrected as

(16) $$t_{sw\_worst}=(1+\frac{2}{R})t_{round}.$$

Finally, the expected latency is the average of (15) and (16):

(17) $$t_{sw}=(0.5+\frac{2}{R})t_{round}.$$

**Figure 20:**
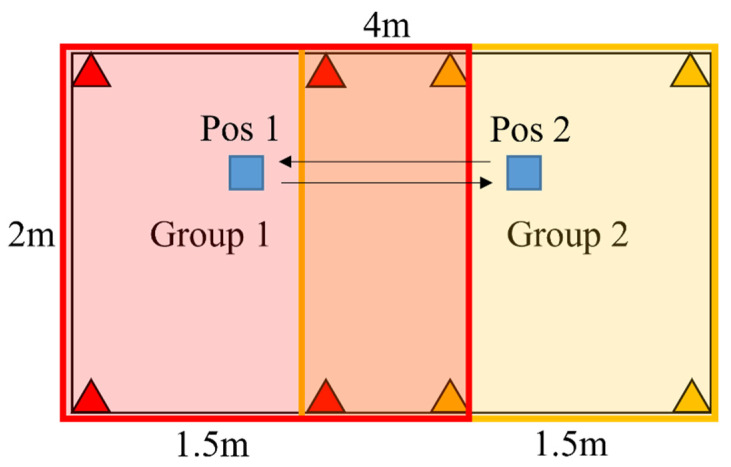
Latency test deployment.

The same deployment as that in Figure 13 is used to verify the inference. The range was 75 ms for each group. The expected latency was 223 ms, as calculated by (17). The positioning regions are depicted in Figure 20, which partially overlap to prevent unstable switching when the tag stays at the junction of regions. The group IDs and regions are programmed into the tag module in advance, and the switching process is implemented. The tag moves back and forth between Pos1 and Pos2 20 times and records the delay between two consecutive outputs with different group IDs. On average, the measured latency was 213 ms, close to the expected value.

## 5. Demonstration

**Figure 21:**
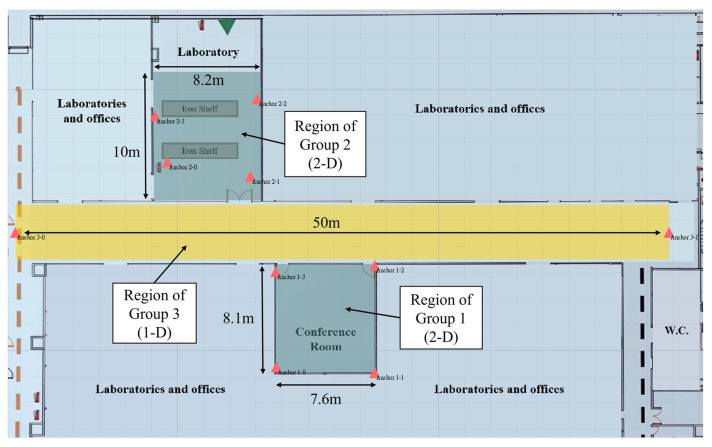
Deployment of groups.

The proposed multigroup positioning system was implemented and tested in a medical center. The positioning area contains a laboratory, aisle, and conference room, as shown in Figure 21. The conference room and laboratory were covered by 2D groups: group 1 and group 2. The aisle was covered by group 3, a 1D group. The red triangles represent the anchors, and the colored areas represent the region of each group. Note that the region of group 3 was unused and defined for better visualization.

**Figure 22:**
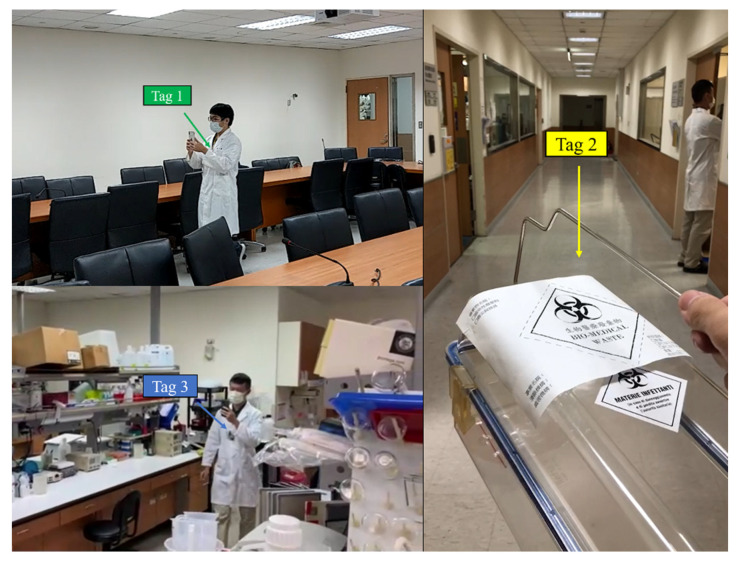
The tags in the experiment.

Due to concerns about the existing medical equipment and the transportation of infectious biological material, the anchors in the laboratory can only be deployed in fixed positions that do not provide full coverage. The tags used in this experiment are shown in Figure 22. Tags 1 and 3 are inside the badges worn by the two researchers. Tag 2 was held by another researcher who was carrying biomedical waste bins for the management of infectious substances.

The 2D groups used DRA-SOT to compute the TDOA position, whereas the 1D group used the algorithm introduced in Section 4.2. The EKF was applied to smooth the trace when tags moved between the groups. The positioning frequency of each group was set to 8 Hz. The positioning timeout was 0.5 s.

**Figure 23:**
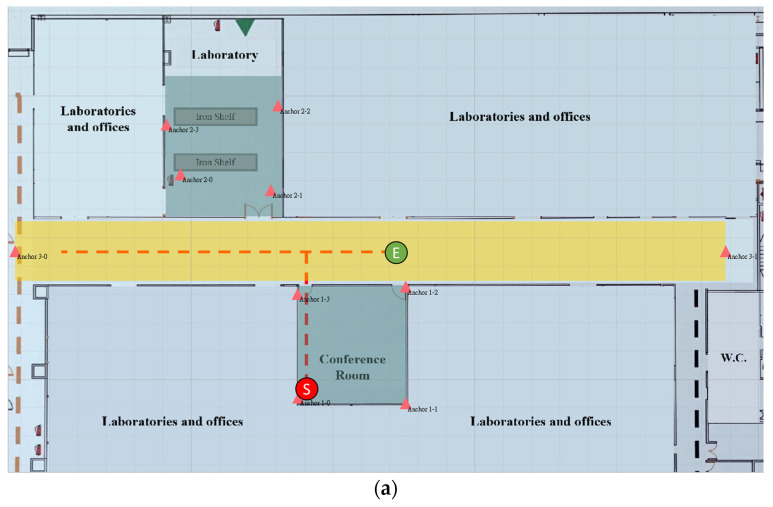

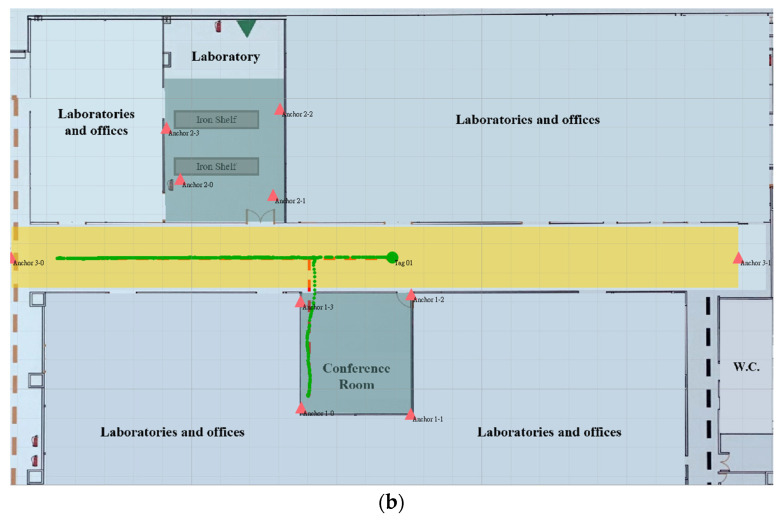
(**a**) Real trace and (**b**) estimated trace of tag 1.

The real trace of tag 1 is shown in Figure 23a. It started in the conference room, moved to the left of the aisle, and finally stopped at the center of the aisle. Figure 23b shows the estimated trace of tag 1 as green dots. The largest dot corresponds to the last position. The total number of collected data was 929, and the positioning RMSE was 28.23 cm. When tag 1 stepped into the aisle, the positions were continuous and accurate because the next desired group could be found using the 2D position estimated in group 1.

**Figure 24:**
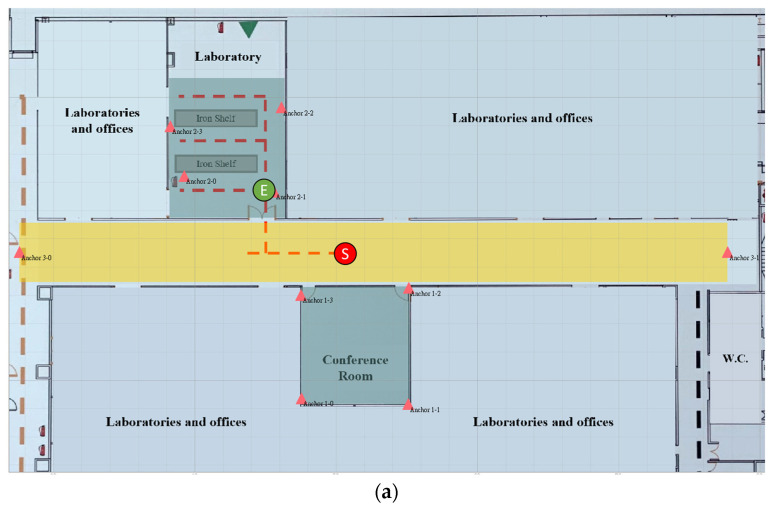

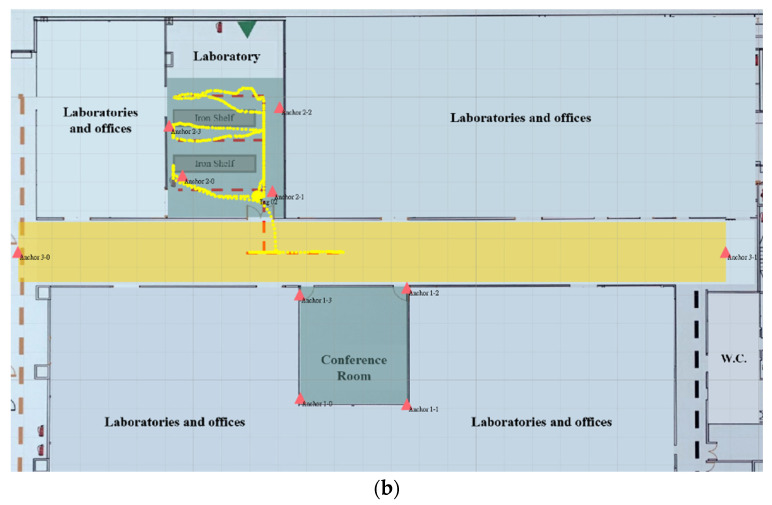
(**a**) Real trace and (**b**) estimated trace of tag 2.

Figure 24a shows the real trace of tag 2. The start and end points are represented by S and E, respectively. It started at the middle of the aisle and moved to the laboratory, which has three rows separated by two working desks with iron shelves. The researcher entered the rows in sequence and came out, standing in front of the door. Figure 24b shows the estimated trace of tag 2 as yellow dots. The total number of collected data was 638, and the positioning RMSE was 45.05 cm. When tag 2 entered the laboratory from the aisle, the measured latency was 1.871 s. Tag 2 could only unbind group 3 in the aisle after a timeout to receive group 2 in the laboratory. The estimated positions in the laboratory rows are scattered because of the severe NLOS errors caused by shelves and the human body.

**Figure 25:**
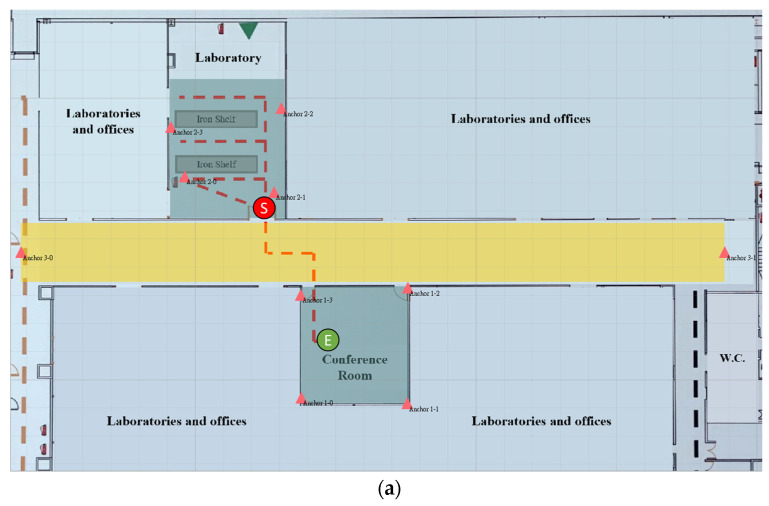

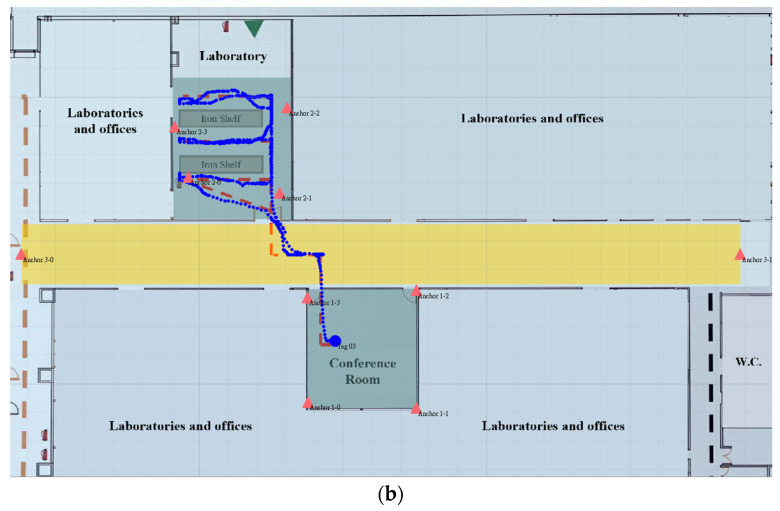
(**a**) Real trace and (**b**) estimated trace of tag 3.

**Figure 26:**
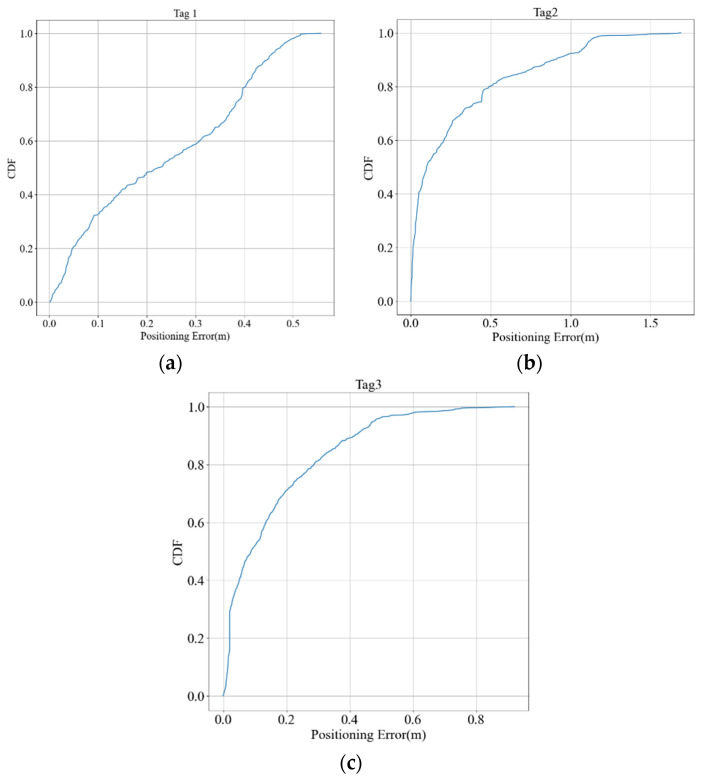
Positioning error CDF of (**a**) tag 1, (**b**) tag 2, (**c**) tag 3.

Figure 25a shows the real trace of tag 3. It started at the laboratory door, entered the rows, and passed through the aisle to the conference room. Figure 25b shows the estimated trace as blue dots. The total number of collected data was 737, and the positioning RMSE was 22.85 cm. The trace of tag 3 is similar to that of tag 2, but its RMSE is lower than that of tag 2. It performed better in the second row in the laboratory and conference room and thus lowered the RMSE. The cumulative distribution function (CDF) of the positioning errors for the three tags are depicted in Figure 26.

## 6. Discussion

### 6.1. Bias in 1D Group

**Figure 27:**
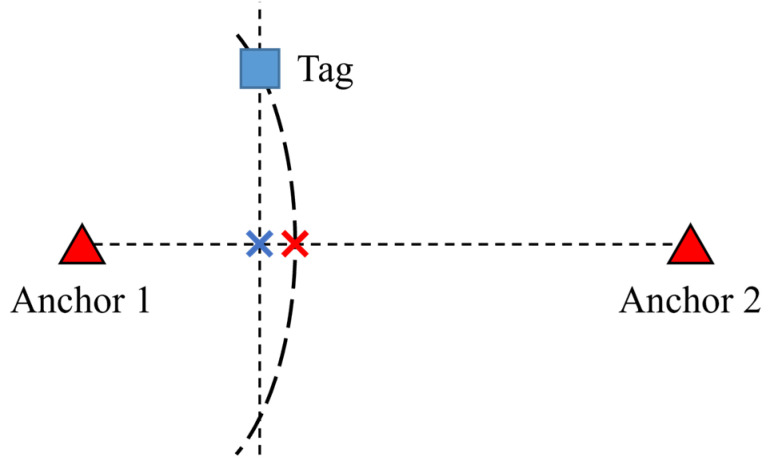
Cause of 1D bias.

The traces from the laboratory to the aisle exhibit a right-bias in Figure 24b and Figure 25b, while the trace from the conference room to the aisle does not show any bias. This can be attributed to the TDOA loss function, as depicted in Figure 27. The ideal estimation for the tag’s position is its projection onto the 1D line marked by the blue cross. However, due to the curvature of the TDOA hyperbola, the intersection with the 1D line occurs at the red cross, resulting in a bias towards the right. This bias increases as the tag move further away from the 1D line. This issue can be resolved by adding another anchor, which is discussed in the next section.

### 6.2. Extended Anchor for 1D Group

**Figure 28:**
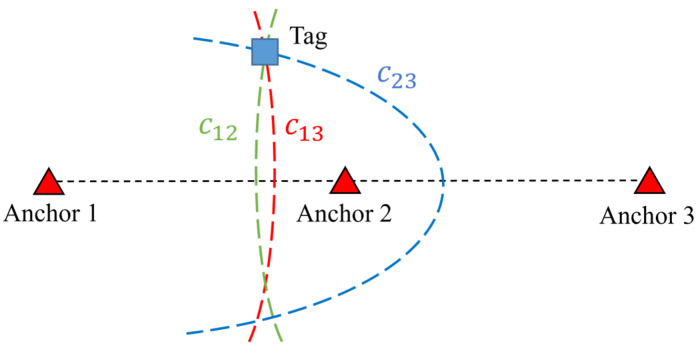
Hyperbolas in a 3-anchor 1D group.

Applying an additional anchor to a 1D group can provide the semi-2D position of a tag, as illustrated in Figure 28. The hyperbolas generated by the three anchors, denoted by $C_{12}$, $C_{13}$, and $C_{23}$, intersect at the tag’s position. Because the semi-2D position can be found, the bias discussed in Section 6.1 does not exist.

**Figure 29:**
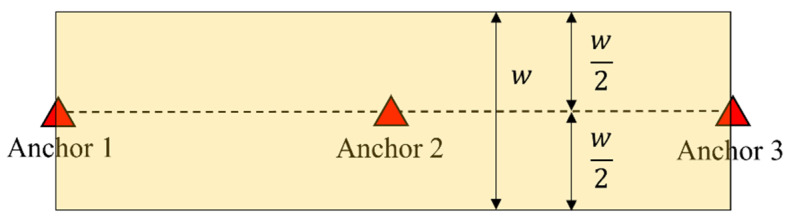
Region of a 3-anchor 1D group.

Furthermore, the region of a 1D group can be defined by its width, as illustrated in Figure 29. By using semi-2D positioning, the tag can determine if it is within the group’s region. If not, the tag can set the BID to 0 and search for other groups instead of waiting for a timeout. This approach can reduce the latency of leaving a 1D group.

## 7. Conclusions

This research paper presents novel algorithms and strategies for expanding the positioning range of a TDOA system. The proposed reference-anchor selection algorithm DRA-SOT enhances accuracy when a tag is positioned beyond the convex hull of the anchors. By selecting an appropriate reference anchor, the local minima of the TDOA loss function around the anchors can be eliminated. Theoretically, the DRA algorithm can be applied to any TDOA optimization method to prevent it from being trapped in local minima. Experimental results demonstrate that the combination of DRA-SOT and EKF can significantly enhance performance in the outside corners of the positioning region compared to methods that do not utilize DRA.

The proposed multigroup system allows for an extended range of coverage in positioning areas where a single group is insufficient. By employing an address-filtering process, the system resolves message conflict problems between groups and improves the likelihood of successful positioning. To facilitate low-latency multigroup roaming, the system incorporates a group-switching process that has been implemented and verified. Furthermore, the proposed 1D group can be utilized in aisle-like environments, thereby reducing anchor deployment costs. A demonstration of the system’s effectiveness was conducted at a medical center, wherein multidimensional groups were employed to cover a laboratory, conference room, and long aisle. The tags were deployed on infectious medical waste and medical researchers to enable security and safety management, and the system accurately located the trajectories crossing the groups. An analysis of the positioning bias and switching latency in the 1D group was conducted, and a potential solution was introduced. This topic is still being investigated and will be further discussed in the future. The proposed multigroup system represents a significant breakthrough in extending the range of coverage for positioning systems. Through the use of an address-filtering process and group-switching mechanism, the system can operate efficiently in complex indoor environments. Furthermore, the application of the 1D group in aisle-like environments offers a cost-effective solution for anchor deployment. The system’s performance was validated through a demonstration that involved tracking tags on infectious medical waste and medical researchers, with the results demonstrating the accurate location of trajectories crossing the groups. The range-extension algorithm and strategies proposed in this paper can also be applied in smart factories, intelligent warehouses, unmanned drone patrols, and other related applications.

## Figures and Tables

**Table 1:** The experimental positioning RMSEs.

Position	Type	SOT + EKF	DRA-SOT + EKF	Improved
A	Outside	0.869	0.376	0.493
B	Outside	0.267	0.272	−0.005
C	Outside	0.365	0.365	0.000
D	Outside	0.243	0.173	0.070
E	Outside	0.570	0.231	0.339
F	Outside	0.170	0.127	0.043
G	Outside	0.554	0.134	0.420
H	Outside	0.077	0.066	0.011
I	Inside	0.048	0.048	0.000
J	Inside	0.053	0.053	0.000
K	Inside	0.060	0.036	0.024
L	Inside	0.038	0.037	0.001
M	Inside	0.090	0.092	−0.002

Unit: meter.

**Table 2:** The number of successful positioning in three testing cases.

Group ID	Binding Group 1	Binding Group 2	No Bound
1	685	0	281
2	0	607	319

**Table 3:** The percentage of successful positioning in three testing cases.

Group ID	Binding Group 1	Binding Group 2	No Bound
1	85.7%	0	35.2%
2	0	76.0%	39.9%

## Data Availability

The data are not publicly available due to privacy concerns.
